# Natural Mineral Water and Diuresis: A Systematic Review

**DOI:** 10.3390/ijerph20085527

**Published:** 2023-04-17

**Authors:** Matteo Vitali, Mario Fontana, Andrea De Giorgi, Daniela Marotta, Serena Crucianelli, Arianna Antonucci, Carmela Protano

**Affiliations:** 1 Department of Public Health and Infectious Diseases, Sapienza University of Rome, 00185 Rome, Italy; 2 Department of Biochemical Sciences, Sapienza University of Rome, 00185 Rome, Italy; 3 Department of Clinical Internal, Anesthesiological and Cardiovascular Sciences, Sapienza University of Rome, 00185 Rome, Italy

**Keywords:** natural mineral water, bottle water, thermal water, diuresis, renal system, systematic review

## Abstract

The present systematic review is aimed at evaluating the diuretic effects determined according to the natural mineral water consumption on healthy individuals. This systematic review has been performed following the guidelines of the PRISMA (preferred reporting items for systematic reviews and meta-analyses) Statement, investigating PubMed, Scopus, Web of Science and Cochrane Library from inception to November 2022. Studies performed both on animals and on humans were considered. After screening, a total of 12 studies have been identified. Of these, 11 studies were performed in Italy and 1 in Bulgaria. The time range of publication is very wide, ranging from 1962 to 2019 for human studies and from 1967 to 2001 for animal studies. All the included studies found an increase in diuresis determined according to the consumption of natural mineral water, in some cases after just one administration of the tested water. However, the quality of the studies is not so high, especially for the research conducted many years ago. Thus, it would be desirable to carry out new clinical studies using more appropriate methodological approaches and more refined methods of statistical data processing.

## 1. Introduction

Natural mineral water, according to the Directive EU 2009/54/EC, is defined as “microbiologically wholesome water, originating in an underground water table or deposit and emerging from a spring at one or more natural or bore exits” [1]. Based on this directive, mineral water can present some indications according to specific criteria, as follows: contains bicarbonate (bicarbonate content greater than 600 mg/L), contains sulphate (sulphate content greater than 200 mg/L), contains chloride (chloride content greater than 200 mg/L), contains calcium (calcium content greater than 150 mg/L), contains magnesium (magnesium content greater than 50 mg/L), contains fluoride (fluoride content greater than 1 mg/L), contains iron (bivalent iron content greater than 1 mg/L), acidic (free carbon dioxide content greater than 250 mg/L), contains sodium (sodium content greater than 200 mg/L). In addition, some mineral waters can be suitable for the preparation of infant food, suitable for a low-sodium diet (sodium content less than 20 mg/L), and they may be laxative or may be diuretic [1]. Additionally, mineral waters can be classified according to their physical and chemical characteristics, such as pH, temperature, and fixed residue at 180 °C. In particular, based on pH, mineral waters can be classified as acid water or alkaline water if they have, respectively, a pH < 7 or >7. Moreover, according to the temperature at source, mineral waters are described as cold (<20 °C), hypothermal (20–30 °C), mesothermal waters (30–40 °C) and hyperthermal waters (>40 °C). Additionally, mineral waters are classified according to the fixed residue at 180°, which indicates the mg of residual mineral salts after the evaporation of 1 L of water at 180 °C. According to the fixed residue at 180 °C, mineral waters are defined as very low mineral content water (<50 mg/L), low mineral content water (50–500 mg/L), medium mineral content water (500–1500 mg/L), and rich mineral content water (>1500 mg/L) [1,2].

The consumption of natural mineral waters can contribute to a proper hydration, even if there is no general consensus on the adequate daily total water intake because body weight, water balance, and plasma osmolality are relevant end points for determining the optimal fluid intake; however, there is a lack of scientific evidence in this field [3,4]. Additionally, natural mineral waters can present one or more properties favourable to health, both in terms of compensation for mineral deficiencies and specific positive effects on different apparatuses. Indeed, drinking natural mineral waters means quenching thirst and ensuring the intake of oligoelements such as calcium, sodium, magnesium, sulphur, bicarbonates, fluorine, which are fundamental, together with water, for the natural course of biochemical reactions and physiological processes [2]. For example, calcium-rich mineral waters are a very relevant source of highly bioavailable calcium with positive effects on bone biomarkers and densitometric parameters [5]. Additionally, an adequate magnesium intake seems to be useful for preventing atherosclerosis, eclampsia and insulin resistance, and for maintaining bone health, consequently preventing osteoporosis [6]. In addition, it is known that natural mineral water can have an essential role in digestive processes, nutrients’ absorption, the elimination of metabolic wastes, and can participate in the proper functioning of the circulatory system [7,8,9,10,11]. Additionally, drinking mineral water unchangeably, with respect to switching to tap water, is associated with a decrease in all-cause mortality in elderly people. Additionally, specific positive effects were demonstrated in older age: drinking natural water rich in calcium is useful to prevent insomnia, osteoporosis and tooth loss; drinking water rich in magnesium seems to protect against atherosclerosis, ischaemic heart disease, arrhythmias, sudden death, and cerebrovascular diseases; drinking sodium bicarbonate mineral water has been related to a significant reduction in total cholesterol, low-density lipoprotein cholesterol, and glycaemia [12].

All these properties are ensured by the stable composition over time of each natural mineral water, as requested by national authorities when recognizing a new mineral water. In addition, more recently, other possible positive effects for human health determined as being caused by natural mineral waters have been evaluated. For example, a study was performed to evaluate the potential therapeutic properties of mineral water in metabolic syndrome and the results suggested that water minimally mineralized and those rich in specific macronutrients (bicarbonate, sulphate and magnesium), in combination with a low-calorie diet, may contribute to control glucose levels and blood lipid in subjects affected by metabolic syndrome [13]. Another study carried out to investigate the possible role of mineral water in intestinal inflammation verified that calcium and magnesium sulphate, sodium chloride, carbonic, and ferruginous water or bicarbonate one present positive effects on intestinal inflammation and the mucosa-associated microbiota [14].

The consumption of mineral water is especially beneficial for the urinary tract, through the several functions pursued by water at this level, with particular consideration for the diuretic effect [15,16]. Mineral waters with specific features can elicit different diuretic effects by triggering peculiar mechanisms. Firstly, it was thought that just the consumption of waters characterized by a low or a very low mineral content were responsible for increasing diuresis; more recently, it was demonstrated that mineral waters with medium and rich degree of mineralization can also have diuretic effects as a result of their specific composition. Indeed, the increase in diuresis following water ingestion does not depend only on the water hypotonia, but also on the presence and relative concentrations of anions and cations characterizing the water itself [17]. Therefore, until a few years ago, only oligo-mineral water was recommended to increase diuresis, while, currently, the guidance is changing and medium or highly mineralized waters are also considered useful for this purpose. However, the most appropriate type of water for increasing diuresis is still under debate. The present systematic review is aimed at evaluating the diuretic effects determined by natural mineral water consumption on healthy individuals.

## 2. Materials and Methods

### 2.1. Selection Protocol

This systematic review has been performed following the guidelines of the PRISMA (preferred reporting items for systematic reviews and meta-analyses) Statement [18]. The review protocol was registered on PROSPERO database (registration number: CRD42021270968).

### 2.2. Strategy of Research

The following bibliographic and citation databases have been screened: PubMed (Medline), Scopus, Web of Science (Science and Social Science Citation Index) and Cochrane Library. The research has been performed using the keywords and MeSH terms using the Booleans operators AND–OR and the following words: “bottle water”, “tap water”, “thermal water”, “drinking water”, “mineral water”, “diuresis”, “diuretic”. The research considered all the articles up to 16 November 2022.

We considered the following inclusion criteria: all the original articles aimed at evaluating the diuretic effects of water consumption, published in any language (further selection including just those written in English or Italian), performed in vivo on animals or on humans. Case reports, case series, letters to editors, commentaries, editorials, critical, systematic review and/or meta-analysis and other articles reporting no new objective data have been excluded from selection. The references cited in critical, systematic review and/or meta- analysis have been screened for further eligible articles. No temporal limits have been set. According to the exclusion criteria, all the articles not responding to the review aim and to the predetermined inclusion criteria have been rejected.

All the references derived from the bibliographic research have been transferred to the reference software Zotero systematic review manager for duplicate removal and the further evaluation of the relevance of each article. Particularly, four investigators (M.V., A.D.G., D.M., C.P.) independently screened all of the potentially eligible studies through the reading of the title and abstract to evaluate the agreement to inclusion criteria. Furthermore, the same investigators independently and integrally read the articles considered potentially relevant. Any disagreement was solved through a discussion among the investigators.

### 2.3. Quality Evaluation of the Studies

At the end of the articles review process, the eligible full texts included seven clinical studies on human beings [19,20,21,22,23,24,25] and five on animals [26,27,28,29,30].

To date, concerning the bias risk evaluation on humans, there is not a universally accepted checklist already used for evaluating the methodological quality of clinical studies focusing on other therapeutic approaches but pharmacological ones. Therefore, following what had already been performed in other systematic reviews dedicated to not pharmacological treatments [31,32,33] in the same specific field [34,35], we used the checklist CLEAR NPT (checklist to evaluate a report of a nonpharmacological trial), for internal validation, which was specifically devised for not-pharmacological clinical trials by a group of experts using the Delphi method [36]. This checklist is built up of 10 questions for which a yes/no/not clear answer is possible. According to what was established by its authors [35], a score ranging from 10 to 8 corresponds to a low risk of bias, a score ranging from 7 to 5 means a medium risk of bias and a score lower than 5 represents a high risk of bias.

The risk of bias for studies on animals has been evaluated using the checklist SYRCLE’s RoB tool (risk of bias tool elaborated by the systematic review centre for laboratory animal experimentation) [37,38]. This checklist is made up of 10 questions investigating the following bias: selection, performance, detection, attrition, reporting and other possible sources. To the 10 answers. a yes/no/not clear answer is possible; any “yes” represents a low risk of bias, any “no” stands for a high risk of bias, whereas any “not clear” states that the reported data are scarce to evaluate the specific risk of bias. In the case of this checklist, as recommended by the researchers who elaborated it [38], an overall score for each study has not been determined in order not to assign a particular importance to a specific dominion which will be difficult to justify.

Four investigators (M.V., A.D.G., D.M., C.P.) independently evaluated the quality of the articles included in the review using the above-described checklists; any disagreement has been solved through discussion.

## 3. Results and Discussion

**Figure 1:**
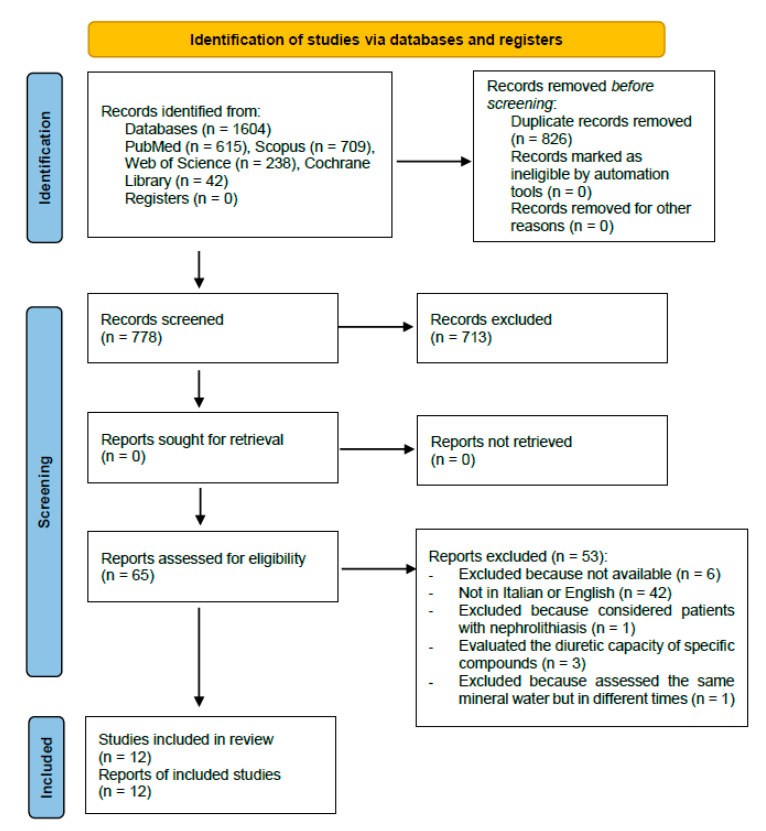
Selection studies flowchart.

Figure 1 shows the details of the review process.

A total of 12 studies [19,20,21,22,23,24,25,26,27,28,29,30], to be included in the review for the qualitative synthesis, have been identified. The initial research on the Pubmed, Scopus, Web of Science and Cochrane databases has allowed us to obtain 1604 bibliographic citations, of which 778 remain after duplicate removal (of which 713 have been excluded because the title or abstract did not satisfy the eligibility criteria). The remaining 65 articles have been fully read and 42 more papers have been excluded because they had published been in different languages that were neither English nor Italian. Initially, no language limitations were set in order not to a priori exclude pertinent articles; however, during the full-text reading, just the articles published in English or Italian have been considered eligible. The former has been selected because of an international level and due to them probably having a better quality; the latter have been selected because they were performed in the country where the mineral water springs cited in the articles are located. Moreover, five more articles have been further excluded because they considered patients affected by nephrolithiasis, considered the diuretic effects of specific compounds or evaluated the same mineral water assumed in different moments. Finally, six articles were not traceable. The references of the selected articles were screened in order to find any further relevant citations; however, no other articles satisfying the inclusion criteria were found. For each article included in the qualitative synthesis of this systematic review, the main investigators extracted the following data: essential bibliographic information, the country in which the study have been set, the eventual source of funding, study design, the type of water studied, the features of the population, patients, and animals that were the object of the study (in terms of race, in case of animals, or gender and age in case of humans), description of the intervention with the studied water, and description of the control intervention (when present), main results, conclusive and original considerations, the overall risk of bias (for studies on humans). These data are summarized in Table 1 (study on human subjects) and Table 2 (study on animals).

As shown in Table 1 and Table 2, 11 of the 12 studies were performed in Italy and 1 in Bulgaria. Indeed, Europe has a leadership position in the production and consumption of bottled water and, among the European countries, Italy is the top producer and consumer, with a per capita yearly consumption of about 200 L and more than 250 producers in 2019 [31,32]. Thus, it is understandable why Italy is the most involved country in studies in this field.

The time range of publication is very wide, from 1962 [19] to 2019 [25] for human studies, and from 1967 [26] to 2001 [30] for animal studies.

Clinical studies enrolling healthy adults and elders are reported in Table 1. The hydropinic intervention, characterized by the consumption of natural mineral water in a unique administration, usually in the morning under fasting, and/or with an administration ranging in duration from one administration [24] to 8 weeks [25]. The outcome is considered as the urinary output before and after the hydropinic treatment or compared to a control treatment performed with drinking, distilled water, isotonic solution or using a different mineral water. All the studies reported a remarkable post-treatment increase in the urinary volume. Additionally, in addition to the increase in urine volume, some studies included in the review also evidenced other indications of diuretic effects. Donnini [19], for example, reported that after the administration of the tested mineral water, the urinary excretion of electrolytes decreased progressively from the first hour onwards until reaching the lowest values at the fourth hour. This low elimination of electrolytes (and consequently of hypotonic urine) is an indirect expression of the diuretic capacity of the tested water, which induces a prolonged inhibition of the mechanism of renal concentration. Mars and Severgnini [20] recovered an increasing solid diuresis, reporting a higher level of azoturia and urinary chloride after the administration of the tested mineral water with respect to that of distilled water. Fiore and Colombrita [21] proved that the urinary-specific gravity was always increased after the administration of the water under study. This increase is probably the expression of an increase in solid diuresis. With regard to the possibility of a loss of serum electrolytes, Messina and Mammarella [22] reported that the blood electrolytes balance (Na, K, Cl, P, Ca) was not significantly modified even with prolonged treatments with a low mineral water. Additionally, Simeoni et al. [23] compared a low mineral water with a calcium bicarbonate alkaline water and found a lower excretion of urinary calcium together with an unmodified oxalate excretion following low mineral water, inducing lower CaxOx products. Nappi et al. [24] evaluated urinary density, azoturia and urate both before the acute loading of water tested and after two hours; the results showed a significant reduction in all the monitored parameters. The five studies carried out on animals, reported in Table 2, were performed on rats, administering a hydropinic treatment mineral water and drinking water, distilled water or a different mineral water as control intervention. The results of an increase in murine urinary volume following the hydropinic treatment confirmed the data obtained in human studies. Moreover, in the study of Evandri and Bolle [30], the urinary volume was not influenced by the mineralization grade of the administered water, highlighting that the low grade of water mineralization is not necessarily the reason for the diuretic effects. To explain these results, it is important to note that mineral water can increase diuresis through different mechanisms. In particular, low mineral waters increase diuresis, eliminate waste products, prevent urinary calculi and have an anti-inflammatory and anti-infective action. Rich in calcium low mineral waters present these last two actions and, according to recent studies, they prevent the formation of urinary calculi [33,34]. The increase in diuresis caused by this type of water is related to the low mineral salts content, particularly of sodium ion, which leads to an osmotic water passage at a tubular level, causing an increase in the urinary output [35]. The medium mineral bicarbonate waters have a diuretic action attributable to hypotonia and to the digesters content. Specifically, the diuretic action is attributable to the presence of alkaline earth metals, acting both on the renal parenchyma, boosting functionality and on the dynamic of urinary tract [36]. A recent study reported that medium mineral water with a high bicarbonate content produces specific urine changes, contributing to the prevention of urinary calculi. Consumption of bicarbonate medium mineral water increases the excretion of uric acid, with no risk of urinary calculi formation due to the increase in the urinary volume, urinary pH and the excretion of citrate [37]. Additionally, carbonic waters can induce an increase in diuresis. This effect is more evident for oligo-mineral or minimally mineralized water; however, carbon dioxide itself, due to the mucosa and stomach vasodilation and the following rapid absorption, presents a remarkable and proper diuretic effect [38]. Finally, rich mineral water may also present a diuretic effect, as demonstrated by a recent study reporting an increase in diuresis after the consumption of 1–2 L of a rich mineral water with a high content of bicarbonate and iron for 10 days [39].

According to the scores produced by the application of the CLEAR NTP checklist to the seven studies carried out on human subjects, these present a high risk of bias and improvable methodological aspects. However, the coherence of the outcomes reported by all the studies analysed allowed the researchers to mitigate the bias burden while strengthening the scientific evidence. Results derived from the application of the SYRCLE’s checklist highlighted some improvable methodological aspects and a certain risk of bias. Even in this case, the coherence of the results of all the studies increases the strength of the evidences.

This review presents some limitations. Firstly, we did not perform a statistical elaboration of the results of the studies included due to the wide variety of the methodological approaches used for conducting each study. However, this systematic review presents an overview of the diuretic effects derived from the consumption of natural mineral waters with different mineralization and composition. Second, the search was carried out from database inception to November 2022 and included articles published from 1962 to 2020. The analytical approach to assess both the characteristics of water and the diuretic parameters has changed over the past decades; consequently, a critical evaluation of the results of each article included should take into account the publication date. Nonetheless, this systematic review provided an overview of the scientific evidences published in this field. Finally, the quality of the studies was not so high; however, all the articles reported similar results in terms of increase in the diuresis after the consumption of tested natural mineral waters.

## 4. Conclusions

The main finding of the present systematic review is that all the included studies found an increase in diuresis determined by the consumption of natural mineral water after just a single administration of the tested water. This evidence emerged both in animal and human studies. Additionally, human studies also reported changes in the levels of other parameters useful for indicating an increase in diuresis, such as the decrease in urinary excretion of electrolytes, higher level of azoturia, urinary chloride and urate, and urinary specific gravity. Despite this, the blood electrolytes balance was not significantly modified even with prolonged treatments with low mineral waters.

However, the quality of the recovered studies in this field is not so high, especially for the studies that were conducted many years ago. Thus, it would be desirable to carry out new clinical studies using more appropriate methodological approaches and more refined methods of statistical data processing.

## Figures and Tables

**Table 1:** Features of studies on human subjects included in the systematic review.

Study; Country Study Design Source of Funding	Type of Water/s Studied	Sample Size	Features of the Population Studied	Treatment	Intervention on Other Groups/Controls	Results and Original Conclusions of the Authors	Quality Evaluation According to the CLEAR NTP Checklist
Donnini 1962 [19]; Italy Controlled clinical study Not reported	Natural mineral water Le Vallicelle, medium mineral bicarbonate–alkaline–earth water	First experiment: 10 healthy human subjects. Second experiment: other human subjects different from those of the first experiment (number not reported)	Not reported	First experiment: 1000 cc per os of Le Vallicelle water in the morning on an empty stomach for one day. Second experiment: 1000 cc per os of Le Vallicelle water all day long for 2 days	First experiment: 1000 cc per os of Le Vallicelle water in the morning on an empty stomach for one day in the same subjects 2 days after the treatment with Le Vallicelle water. Second experiment: 1000 cc per os of Le Vallicelle water all day long for 2 days.	First experiment: Increase in the urinary volume during the first hour after the administration of Le Vallicelle water compared to equal volume of drinking water. Second experiment: Increase in the urinary volume in the 24 h of the 2 considered days during the administration Le Vallicelle water compared to drinking water.	3 Yes, 6 No, 1 Unclear High risk of bias
Mars and Severgnini 1966 [20]; Italy Controlled clinical study Not reported	Natural mineral water Val Masino medium mineral sulfate–alkaline–silicic water	20 healthy human subjects	10 males (20–40 years old) and 10 males (70–85 years of age)	1000 cc per os of Val Masino water in the morning on an empty stomach for one day	1000 cc per os of distilled water in the morning on an empty stomach for one day in the same subjects 5 days after the treatment with Val Masino water.	Increase in the urinary volume during the administration of Val Masino water compared to distilled water (statistically significant results in the group younger group of subjects).	3 Yes, 4 No, 3 Unclear High risk of bias
Fiore and Colombrita 1975 [21]; Italy Controlled clinical study Not reported	Natural mineral water Currone, medium mineral bicarbonate–alkaline–earth water	10 healthy human subjects	10 males (22–35 years of age)	600 cc per os of Currone water in the morning on an empty stomach for one day in the same subjects 2 days after the treatment with drinking water	600 cc per os of drinking water in the morning on an empty stomach for one day.	Statistically significant increase in liquid diuresis in the 4 h following the administration of Currone water respect to drinking water. Increase in the urinary specific weight of the specimen s collected in the 4 h after the administration of Currone water respect to drinking water connected to the increase in solid diuresis.	2 Si, 7 No, 1 Unclear High risk of bias
Messina and Mammarella 1983 [22]; Italy Controlled clinical study Not reported	Natural mineral water Levissima low mineral bicarbonate–alkaline–earth water	Not reported	Not reported	1000 cc per os of Levissima water	1000 cc per os of saline solution isotonic respect to Levissima water.	Increase in the diuresis during the 3 following hours after the administration of Levissima water respect to saline solution (>16%).	1 Yes, 8 No, 1 Un clear High risk of bias
Simeoni et al., 1998 [23]; Italy Controlled clinical study Not reported	Natural Mineral Fiuggi water; low mineral calcium bicarbonate water	21 healthy human subjects	11 men (39 ± 3 years) and 10 women (37 ± 4 years)	2000 cc per os of Fiuggi water during the day	2000 cc per os of calcium bicarbonate-alkaline water during the day.	Increase in the diuresis following the administration of Fiuggi water respect to control water.	3 Yes, 1 No, 6 Unclear High risk of bias
Nappi et al., 2003 [24]; Italy Controlled clinical study Not reported	Natural mineral Ielo water; low mineral calcium bicarbonate water	30 healthy human subjects	Group 1: 16 males e 4 females (61.9 ± 15.7 years). Group 2: 5 males and 5 females (53.0 ± 12.5 years)	First experiment: 500 mL per os in 15 min of Ielo water in the morning on empty stomach. Second experiment: 1500 mL per os of Ielo water/day for 10 consecutive days	First experiment: 500 mL per os in 15 min of drinking water in the morning at empty stomach for one day. Second experiment: 1500 mL per os of drinking water/day for 10 consecutive days.	First experiment: increase in the urinary volume 60 and 120 min after (statistically significant at 120 min) after the administration of Ielo water respect to controls. Second experiment: significant increase in the urinary volume following the administration of Ielo water respect to controls.	3 Yes, 1 No, 6 Unclear High risk of bias
Roussev et al., 2019 [25]; Bulgary Not Controlled clinical study Bulgarian National Research Fund	Natural medium mineral Varna water; sulphate-alkaline-earth water	50 healthy human subjects	7 men and 43 women (40-65 years)	20 mL pro Kg of body weight for 24 h every day for 8 weeks consecutively	-	Statistically significant increase in the urinary volume after 8 weeks compared to before the treatment with Varna water.	2 Yes, 7 No, 1 Unclear High risk of bias

**Table 2:** Features of animals’ studies included in the systematic review.

Study; Country Study Design Source of Funding	Type of Water/s Studied	Sample Size	Features of the Population Studied	Treatment	Intervention on Other Groups/Controls	Results and Original Conclusions of the Authors	Quality Evaluation According to the SYRCLE’s Checklist
Brunelli 1967 [26]; Italy Study on animals Not reported	Natural Mineral San Pellegrino water, medium mineral sulphate-alkaline-earth water	Not reported	Male rats, of the Sprague Dawley strain	Spontaneous assumption of San Pellegrino water in a metabolic cage for 15 days in autumn and 10 days in winter	Spontaneous assumption of distilled water (group 1) or spontaneous assumption of drinking water (group 2) in a metabolic cage for 15 days in autumn and 10 days in winter.	The spontaneous assumption of distilled water did not determine any diuretic effect, indeed rats having distilled water had lower urinary output in winter months, compared to liquid assumption respect to San Pellegrino or drinking water.	3 Yes, 3 No, 4 Unclear
Federici and Pasqualis 1984 [27]; Italy Study on animals Not reported	Natural mineral Tesorino water, medium mineral bicarbonate-sulphate-earth water	78 rats (40 with Tesorino water and 38 with distilled water)	Female albino Wistar M. rats	25 mL pro kilo rats of Tesorino water administered using a nasogastric tube	25 mL pro kilo rats of bi-distilled water administered using a nasogastric tube.	Statistically significant increase in diuresis after 6 and 24 h followed by the administration of Tesorino water respect to bi-distilled water.	4 Yes, 2 No, 4 Unclear
Federici 1985 [28]; Italy Study on animals Not reported	Waters with different mineralization	6000 tests on rats	Female Albino Wistar Rats S.M	Waters with different mineralization	Drinking water of Parma aqueduct or distilled water	Increase in the diuresis following the administration of mineral water respect to drinking and distilled water; increase in diuresis following the administration of drinking water respect to distilled water.	4 Yes, 5 No, 1 Unclear
Federici et al., 1988 [29]; Italy Study on animals Not reported	Waters with different mineralization	12,000 tests on rats	Female Albino Wistar Rats S.M.	Waters with different mineralization	Waters with different mineralization	Increase in the diuresis following the administration of medium mineral water respect to low mineral ones.	4 Yes, 6 No, 0 Unclear
Evandri and Bolle 2001 [30]; Italy Study on animals Not reported	Natural mineral Waters with different mineralization	120 rats	Male Albino Wistar Rats S.M.	25 mL pro kilo/rat of water with different mineralization water administered using a nasogastric tube	25 mL pro kilo/rat of drinking water administered using a nasogastric tube	Diuresis is not influenced by the mineralization level of tested waters.	3 Yes, 4 No, 3 Unclear

## Data Availability

The data are contained within the article.
